# Design, Kinematics and Gait Analysis, of Prosthetic Knee Joints: A Systematic Review

**DOI:** 10.3390/bioengineering10070773

**Published:** 2023-06-27

**Authors:** Faiza Rasheed, Suzanne Martin, Kwong Ming Tse

**Affiliations:** 1 Department of Mechanical Engineering and Product Design Engineering, Swinburne University of Technology, 3122 Victoria, Australia; frasheed@swin.edu.au; 2 Institute for Health and Sport, Victoria University, 3011 Victoria, Australia

**Keywords:** artificial knee unit, knee prosthesis, prosthetic knee joint, transfemoral leg amputation, transfemoral amputee kinetics, knee kinematics, gait analysis, knee biomechanics

## Abstract

The aim of this review article is to appraise the design and functionality of above-knee prosthetic legs. So far, various transfemoral prosthetic legs are found to offer a stable gait to amputees but are limited to laboratories. The commercially available prosthetic legs are not reliable and comfortable enough to satisfy amputees. There is a dire need for creating a powered prosthetic knee joint that could address amputees’ requirements. To pinpoint the gap in transfemoral prosthetic legs, prosthetic knee unit model designs, control frameworks, kinematics, and gait evaluations are concentrated. Ambulation exercises, ground-level walking, running, and slope walking are considered to help identify research gaps and areas where existing prostheses can be ameliorated. The results show that above-knee amputees can more effectively manage their issues with the aid of an active prosthesis, capable of reliable gait. To accomplish the necessary control, closed loop controllers and volitional control are integral parts. Future studies should consider designing a transfemoral electromechanical prosthesis based on electromyographic (EMG) signals to better predict the amputee’s intent and control in accordance with that intent.

## 1. Introduction

The emergence of rehabilitation has enabled amputees to regain their mobility by using various types of prostheses. Transfemoral amputees have their legs amputated between the hip and knee, at the level of thigh muscles [1,2]. Active or powered prostheses are externally powered through different kinds of motors and function accordingly [3]. They offer ameliorated efficiency and gait, yet they are very complicated [4,5]. On the other hand, most passive knees serve the simplest purpose of primary usage, i.e., to walk at ground level [6]. They normally have hydraulic or pneumatic components that provide fixed impedance, and hence unable to handle locomotion under different situations. They do not offer control through sensors to monitor the interaction between the user and the environment. Moreover, they do not have an external power source, for a control system to be used [7,8,9]. The current passive commercially available prototypes provide walking stability only for level ground; however, they must be considered for uneven terrain as well, particularly in developing countries [10]. Although some prostheses have been created as hybrids, which can function as passive or active prostheses, they offer less stability [11]. Sometimes, the use of a transfemoral prosthetic leg causes extra fatigue for the other healthy limb.

Standards for a microprocessor-based prosthetic knee were developed by Ottobock C-leg. While carrying a burden, it maintains body movements and prevents falls [12,13,14]. Both passive and microprocessor-based prosthetic knees exhibit a rise in midstance and push-off work for the intact leg with increased walking speed, but this rise has no effect on the prosthetic limb. As a result, a prosthetic limb nearly completely loses energy and is dependent on the intact limb for energy changes [15]. The efficiency of healthy limbs may eventually be impacted by this additional effort. Due to the additional labor being done on the prosthetic limb, its physiology may soon begin to deteriorate. Due to this dependence, research is needed to increase the prosthetic knee joint’s effectiveness over time without causing extra load on the intact limb [16,17].

The increased risk of tripping with transfemoral prostheses is another problem. Tripping incidents are common among people using transfemoral prostheses. De facto, transfemoral prostheses increase step width while improving gait symmetry and energy efficiency. To reduce the step width, a specific strategy must be designed [18,19]. The step width problem can be solved with voluntary control. For many upper limb actions, complete volitional control mechanism-based prostheses are now in existence; however, comparable lower limb prostheses are currently lacking. There is further work to be done on an EMG-based transfemoral prosthesis with improved stability, full control over ambulation, transition activities, and symmetrical gait patterns [20,21,22]. Ambulation on an uphill continues to be a difficulty for researchers and requires further investigation.

Numerous prostheses have been created that successfully offer full kinematics and kinetics for walking on level ground and climbing stairs [23]. However, the biomechanics of ramp walks are frequently neglected [24]. To increase the effectiveness of a transfemoral prosthesis, work must be done on the inclined surface kinematics, which is presently lacking. An electromechanical transfemoral design, providing the required energy, torque, and necessary impedance control, based on intent recognition, can help resolve these problems.

The main goal of this systematic review is to offer a critical evaluation of the functions of the knee prosthetic units already in use, as well as an assessment of their levels of mobility, ranges of motion, degrees of freedom, dependability, confidence level, independence, and overall standard of living for amputees. Second, their flaws or deficiencies are evaluated as well, including the risk of falling, ease of mobility and use, design, gait, and kinematics. This systematic evaluation also examines how closely or how differently these transfemoral prostheses functioned naturally compared to a knee joint.

## 2. Materials and Methods

In this study, Preferred Reporting Items for Systematic Reviews and Meta-Analyses (PRISMA) guidelines were followed [25].

### 2.1. Inclusion Criteria and Selection Process

There were several transfemoral prostheses used, with a focus on the role of the knee unit in gait biomechanics. Transtibial prosthesis-related studies, osseointegrated prosthesis, and those that ignored gait biomechanics were disregarded. The outcomes included mobility, running, supporting body weight, preventing falls, weight, durability, cosmesis (appearance), comfort, wear-and-tear, and pain [26,27]. The prosthetic knee joint, type, model, together with its gait on flat and uneven surfaces, stair ascent and descent, ramp walk, and transition activities from sitting to standing and standing to sitting, were all thoroughly documented [28].

**Figure 1:**
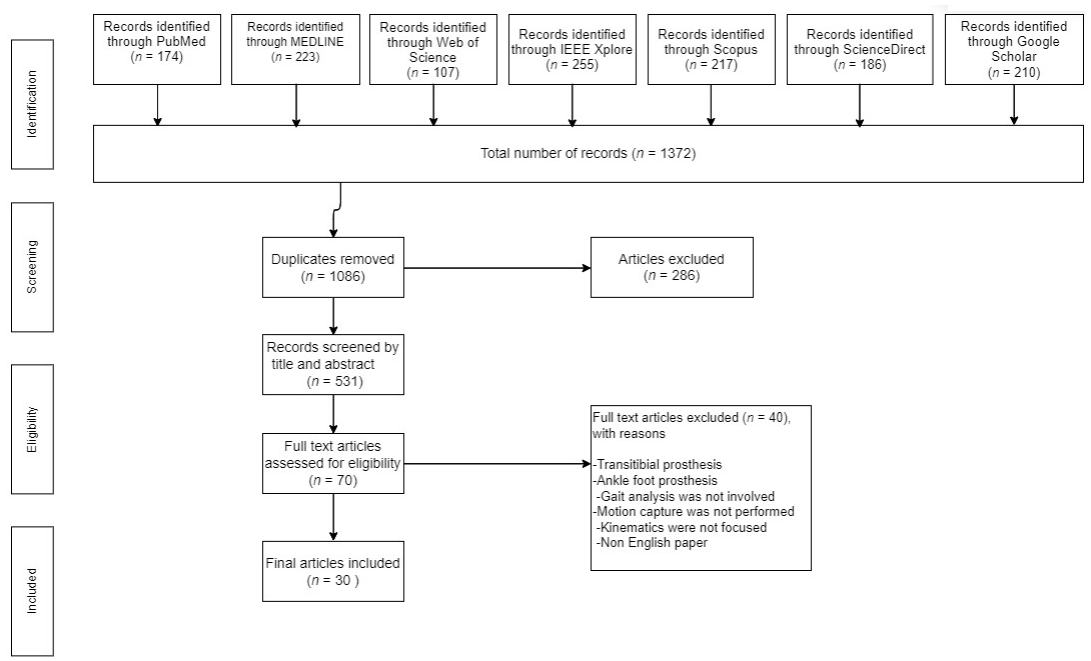
PRISMA flow chart for study process.

Duplicates were removed and titles and abstracts were screened at level 1, and entire texts were screened at level 2, using Covidence and Endnote [29,30]. If the targeted group was not transfemoral amputees or if the research focus was something other than design, kinematics, and gait, the title and abstract that did not fit these inclusion criteria were excluded. As indicated in Figure 1, at level 1 the causes of exclusion were noted.

### 2.2. Search Strategy

The search template for MEDLINE was created using rehabilitation-related terms and keywords including transfemoral amputation, gait analysis, knee kinematics, and prosthetic knee unit design. The same method was then applied to other databases.

### 2.3. Data Collection Process

Data was extracted, and its accuracy was then evaluated. Author, year, region, study group, level of amputation, kind of prosthesis, degrees of freedom, range of motion, research methodology, outcome measures, and key findings were obtained.

### 2.4. Study Risk of Bias Assessment

Using the Cochrane “risk of bias” technique, the risk of bias in studies was independently evaluated [31]. PRISMA criteria were adhered to manage any potential bias risk. The risk of bias was evaluated after each study underwent an independent examination at levels 1 and 2. No articles by any of the authors were used in this systematic review.

## 3. Results

### 3.1. Attributes of Selected Studies

From our search, 1372 citations in all were located. After eliminating duplicates (*n* = 286), at level 1 we examined 1086 citations. 30 publications were found after two screening steps were completed at level 2: one involved titles and abstracts and the second involved entire articles (Figure 1). Every study was conducted in English and all adult community members who had transfemoral amputations were included. Male respondents made up 78% of the studies’ sample population, and they ranged in age from 21 [32] to 62 [33] years on average. Two research [34,35] used virtual environments, and the sample sizes ranged from *n* = 1 to *n* = 20. In 22% of the studies, comparisons with healthy subjects were made.

### 3.2. Microprocessor-Based Prostheses

Studies show that microprocessor-controlled prosthesis normally performs better than non-microprocessor-controlled prosthesis. A comparison of novel microprocessor-controlled prosthetic knee (MPK), named I-knee, was developed with non-microprocessor-controlled prosthetic knees (NMPKs), for its efficiency at different walking speeds [36]. The comparison of gait symmetry and peak knee flexion angles during swing phase, showed that I-KNEE is more robust to speed alterations than NMPKs, making it more effective in speed-varying settings. Moreover, Stance-control swing-assist (SCSA) knee prosthesis addressed the passive stance-controlled microprocessor-controlled knees’ (SCMPK) low output impedance in the swing mode [37]. It’s closed-loop control system allowed variations from the typical swing phase, tranquil operation and an inertia-driven swing phase, which overall ameliorated swing-phase characteristics. These comparisons direct us towards a transfemoral prosthesis offering complete control for different ambulation activities.

### 3.3. Design

Various mechanical and electromechanical prostheses have been developed so far, which work to fulfill requirements regarding biomechanical parameters. However, few are commercially available, mostly are under research in laboratories [32,33,34,38,39]. A locking mechanism restricted amputee’s fall during running by preventing undesired knee flexion [32]. A one-way clutch allowed knee extension only when the prosthetic knee was loaded. In likely research, prosthesis used time as a controlling factor rather than the conventional approach of using ground reaction forces to lock and unlock the knee during flexion [38]. Running safely was made possible by the mechanical design that only allowed the knee to flex during the first half of the swing phase. However, these passive designs could not focus on the torque and power requirements for ambulation. A current hybrid prosthetic knee combined a spring-damper system, electric motor, and transmission system to effectively ambulate stairs [39]. The reworked actuator design reduced prosthesis weight and ensured the necessary torque and power in active mode. Similarly, hybrid prosthesis presented by Bartlett et al. [33] had some active power when required, but for the most part it behaved passively during the swing phase. The system design included back-drivable linear electromechanical drive system, a new actuator, and a hydraulic cylinder. The passive mode lacked required energy for ambulation. Moreover, a novel over-actuated knee prosthesis succeeded to control speed and torque according to the various types and phases of locomotion [34]. The mechanical design had two motors: one high speed/low torque motor controlled swing phase, and the other low dynamics/high torque motor made possible the completion of tasks with required active torque. This system did not cover stance phase of gait. Therefore, an electromechanical prosthesis can address both swing and stance phases of gait to offer natural walking.

### 3.4. Biomechanical Parameters

Various research studies have been carried out on transfemoral prosthesis, which meet biomechanical parameters to different extent, as represented in Table 1. The prosthetic knee, known as the Utah knee, employed an actively variable transmission (AVT) system that optimized peak knee velocity in swing phase, knee angle, torque, and power trajectories for stair ambulation and ground-level walking [21]. However, ramp walk was not their focus. Mechanical Knee-Ankle-Toe Active Transfemoral Prosthesis (KATATP) produced the necessary torque and required hip, knee, ankle, and toe angles after analyzing various gait phases [40]. This model helped amputees obtain more natural and regular walking gaits, however real time implementation was left. On the other hand, Wang et al.’s [41] prosthetic knee joint assessed the biomechanical traits by deploying a motion acquisition and analysis system along with three 3D force measuring plates. Subject position was acquired by gathering marker movement information. They monitored the ground reaction forces, knee and ankle joint angles, and peak knee torque patterns, using Mokka tool. However, the service life was compromised at the end. Rahmi et al. [42] generated comparison for mechanical and pneumatic four bar linkage prosthetic knee joints regarding the energy expenditure associated with walking. They found out that pneumatic system showed lower energy expenditures and faster walking. Ultimately, an utmost control for biomechanical parameters can be achieved with a controller-based prosthesis, through feedback system.

### 3.5. Stairs Ambulation

After ground level walk, next goal for researchers is the stairs ambulation. Tran et al. [21] devised lightest fully powered knee prosthesis offered the required broad range of torque and speed for both stair climbing and level ground walking. The redesigned actively variable transmission (AVT) along with the Utah knee encompassed locomotion on stairs with necessary power. And a three-level control system facilitated various ambulation modes, except ramp walk. An adaptive control knee prosthesis was proposed for stair climbing and ensured adjustment to different stair heights, cadences, and gait patterns [43]. It’s position controller supplied necessary knee and ankle joint angles, based on the subject’s thigh movement during the swing phase. Another controller designed for stair ascent was also based on amputee’s residual thigh motion [44]. It’s new phase variable enabled participant to ascend stairs by replicating healthy subject kinematics along with net positive mechanical work. However, it could not provide volitional control. Similarly, a lightweight, active transfemoral prostheses for walking and stair climbing were presented by Hood et al. [45]. Their kinematic and kinetic investigations produced controlled weight acceptance, forward propulsion, and swing clearance and quantified differences between active and passive prosthesis. However, a transfemoral prosthesis capable of stairs ambulation along with volitional control is still lacking.

### 3.6. Ramp and Uneven Surface Walk

Various prostheses offering ramp walk have been the focus of researchers yet require realization for commercial availability. Azimi et al.’s [46] research monitored knee position, velocity, and torque for uneven surfaces. An intended stable gait was validated by three controllers. Another active prosthesis modeled knee joint kinematics for ramp walk and stair ascent, including steady-state and transitional gaits [46]. Both steady-state models characterized human ambulation as a function of features like gait phase, forward speed, and slopes, while both transition models combined two steady-state models with a conditional offset. Simulation outcomes depicted the model’s capability for adaptive slope identification and mode classification errors. Sturk et al. [35] highlighted stable gait over level, sloped, and uneven terrains. Participants’ walks over ground, inclined, and uneven surfaces were recorded in a virtual environment, and their various parameters like medial-lateral margin-of-stability (ML-MoS), step strategies, and gait changeability were compared with healthy subjects for gait adaptation. A powered above-knee prosthesis produced natural walk on inclined surfaces, however, devoid of any estimation of the incline ahead [48]. It’s control framework monitored impedance management during stance and trajectory tracking during the swing phase. A proportional-derivative (PD) controller checked knee joint angle and knee trajectory, for the swing phase, to cope with real-time ramp walk challenges. Controlled ramp walk along with intent prediction to better handle speed and impedance still needs to be worked on.

### 3.7. Gait Patterns

Gait patterns for various terrains, with various walking speeds and switching over have been investigated by researchers. Andrysek et al. [49] appraised gait patterns linked with two styles of mechanical stance control prosthetic knee units—a weight-activated braking knee and an automatic stance-phase lock knee. Investigation of kinematic and kinetic parameters showed that weight-activated braking knee had prolonged swing-phase duration, a higher range of motion, earlier ankle push-off, and greater anterior pelvic tilt. Moreover, two distinct algorithms for intermittent and continuous walking created command signals for the knee and ankle joints, as well as a transition strategy from one method to the other [50]. They followed knee accelerations, number of steps taken, knee and ankle joint references, gait phase, and detected mode of the prosthesis to produce requisite command signals for smooth switching as per requirement. Additionally, gait features of two common types of friction-based swing-phase controlled prosthetic knee units were analyzed [51]: first was constant-friction (CF) and second one a variable cadence controller (VCC). A 2D motion analysis set-up computed gait parameters including walking velocity, swing-phase time, cadence, stride length, step length, double support, and knee flexion. VCC ameliorated various gait patterns linked with prosthetic swing-phase control, including swing-phase timing and peak knee flexion angles. These prostheses ensured good kinematics, however, could not offer the merit of volitional control, to improve the efficiency of prosthesis.

### 3.8. Impact of Materials on Prosthesis

Normally different types of materials are used for manufacturing prosthetic knees for transfemoral amputees, including aluminum, titanium, biomaterials like fiber reinforced composites, glass fiber composites etc. [12,61,62]. The best composites for passive prosthetic limbs are Carbon fiber-reinforced polymer (CFRP) owing to the flexibility and reduced weight merits which combinedly offer comfort, higher strength and stiffness, as compared to other biomaterials [61]. These composites allow for energy storage to provide power to body for performing various actions. Polycentric prosthetic knee mechanism provides more comfortable and stable gait phases, as compared to single axis knee designs [62]. For the manufacturing of polycentric knee, three materials were compared: aluminum, CFRP (carbon fibre-reinforced plastics) and GFRP (glass fibre-reinforced plastics). The comparison was developed on the basis of improved mid-swing toe-clearance for safe walk on uneven surfaces, low maintenance, high endurance against fatigue loading and minimal metabolic energy expenditure. FEA (finite element analysis) results proved CFRP as a best material in terms of providing higher fatigue endurance as compared with other two materials. Another indigenous prosthetic knee design was made successful by incorporating lightweight aluminium 7075 T6 alloy [12]. It showed lesser deformation, reduction of weight and an improved, feasible factor of safety. Fatigue simulation outcome showed a life span of at least 10 years, which validated its safe design. Along with cost reduction, it offered ameliorated stability and kinematics.

## 4. Discussion

In the systematic review, we screened various transfemoral prostheses addressing different biomechanical parameters for movement, to find a research gap. Firstly, this review found multiple aspects, covered in different research articles. For instance, microprocessor-based and non-microprocessor-based prosthesis, gait analysis, kinematics, prosthesis designs, types of ambulation activities, control strategies, volitional control, etc. A comparison of microprocessor-based prosthetics with non-microprocessor-based prostheses proved that microprocessor-based prostheses were more efficient and intelligent, to ensure controlled movement of the knee joint for a stable walk [36,37]. However, the desire for control over ambulation activities ultimately led to active prostheses with particularly designed controllers. Devoid of these closed-loop systems, stable walking along with desired control is not feasible [24,40,44].

Most of the researchers have preferably worked on stairs ascending and descending and offered required biomechanics, however, could not address intent recognition to adjust to stair height, cadence, etc. [24,44,45]. Some research was particularly based on inclined surface locomotion [46]. However, it was just simulated for the robustness test and lacked GRF impact to take into consideration. Likely, a study tested prostheses on healthy subjects, which would cause modified biomechanical parameters in amputees [46]. Additionally, they deployed average values for kinematic data testing rather than subject-specific data. Its real-time implementation could involve environmental interaction, and the introduction of a continuously varying impedance controller could help deal with joint kinetics. Similarly, testing in virtual environments is another issue that could not consider issues faced in real-time implementation [35].

To provide desired control, active prostheses require closed-loop systems with particularly designed controller(s). Some controller-based studies validate their prosthesis efficiency for stable gait in just simulation [46]. However, their real-time experimentation is missing which could consider unexpected scenarios such as push or hindrance as well. Another deficiency is performing real-time testing for stair ascending, while considering GRF. To address these flaws real-time scenarios are needed. The negative damping could be used to generate the required energy in active prosthesis [52]. De facto, this was the first instance of an active prosthetic knee that could operate on its own power, in a subject experiment, for electrical energy regeneration. Prior training and a better experience with the prosthesis always assist amputees to better adapt to the provided prosthesis [53]. Likely, experimentation with a larger set of subjects would help to generalize the working of prostheses and make them adaptive.

Research work for transfemoral prosthetic knee joint led to the point that volitional control and surface recognition are interlinked, as the variation in the surface would make amputees make accordingly decisions [54,55,56,57,58,59]. Different algorithms are deployed for surface electromyography (sEMG), for instance, recurrent neural network (RNN), convolutional neural network (CNN), support vector machine (SVM), etc. EMG-based prostheses are gaining acceptance, owing to increasing control. A single-channel EMG signal-based surface identification method uses a single classifier [54]. Their results could be promising in the future, if tested in a real-time scenario, rather than a virtual environment [55,56,57]. Similarly, some EMG-based algorithms are tested offline, rather than with actual prostheses and amputees [55]. They were worn by able-bodied subjects, to capture EMG signals and testing. The maximum muscle contraction would be different with amputees, depending on their remnant thigh muscles. Hence, accordingly different EMG data would be generated, which requires processing accordingly. Most research articles focus on anticipating knee joint angles and various methods have been developed for this purpose, which use sEMG [58]. The requirement is to constantly monitor and predict motion variables of amputees to ensure a nice control of the prosthesis. This is a requirement in the field of rehabilitation and robotics as well. Devoid of this, the prosthesis fails to provide a satisfactory efficiency level. Most studies focus on the prediction of continuous and smooth locomotion; however, different locomotion modes such as stair and ramp ascending and descending require anticipation [59,60]. Environment perception based on vision could help to predict uneven surfaces. This would ameliorate prosthesis functionality in complex scenarios.

For the manufacturing of prostheses, material selection is very crucial. Various factors are considered for material selection, out of which strength, stiffness, and cost are among the most important ones [61,62]. Owing to the higher merits offered by biocomposites for the manufacturing of passive prostheses, the requirement is to reduce their cost by deriving them naturally and making them cost-effective [61]. This will ensure the provision of more biocompatible, biostable, and eco-friendly material for prosthesis, offering strength, stiffness, low maintenance, long life, durability, non-corrosive nature and high load endurance. In a comparison of aluminum, CFRP (carbon fiber-reinforced plastics), and GFRP (glass fiber-reinforced plastics), for polycentric prosthetic knee designs, CFRP showed the higher fatigue loads endurance, lighter weight, long life, and reduced replacement costs merits [62]. However, for an active transfemoral prosthesis, CFRP is not a favorite material owing to its poor electrical conductivity.

## 5. Conclusions

This systematic review has analyzed current state-of-the-art research on different types of design and biomechanical features of transfemoral prostheses. The reviewed articles present evidence for our hypothesis that active prostheses have much more pronounced results as compared to the passive ones regarding the kinetics and kinematics of the prosthetic knee joint. The articles based on gait analysis and biomechanical parameters have shown the reliance on closed-loop control systems along with power sources. Likely trained amputees show vigorous amelioration in results as compared to untrained amputees. Hence, amputees require appropriate training time prior to using prostheses, either for testing or personal usage.

Researchers in the field of transfemoral prosthesis are recently focusing on electromechanical design and control of powered prostheses; however, there are still many areas that need to be emphasized. Firstly, modern controlled prostheses designed for stable walking are still limited to research and experimental work and are not yet commercially available owing to their higher cost. Most prostheses are limited to model frameworks, real-time testing is missing, or their experimentation is performed just on healthy subjects in a virtual environment. A control system for a prosthetic knee unit requires work to ensure a comfortable, stable, and free-of-fall walk, with real-time testing with transfemoral amputees. More realistic results would be obtained when experiments were conducted on large groups of varied participants and on amputees.

Regarding different ambulation activities, various researchers have focused on ground-level walking and stair ambulation with ameliorated performance. The challenge is to focus on the kinematics and gait analysis of the transfemoral prosthesis for ramp walking to ensure the required symmetrical gait pattern, knee joint angle, torque, power output, and impedance control without causing load on the hip and other healthy limbs. This would require controlled speed for ascending and descending, particularly to prevent falling during descent.

Adaptation to various locomotion modes, gait phases, and uneven surfaces is dependent on anticipation methods, and EMG plays an elementary role in this requirement. Since EMG provides intent recognition to transition between ambulation modes and complete control to walk over different types of surfaces with different speeds and gait cycles. However, active EMG-based transfemoral prostheses are still a challenge for researchers and need attention.

## Figures and Tables

**Table 1:** Summarized details of the studies incorporated in this review.

Authors (Year)	Sample Size	Study Objective (s)	Methods	Outcome Measures	Key Findings
Tran et al., 2019 [24]	1	To develop first completely active knee prosthesis that is light weight and provides required torque and speed for level ground and stairs ambulation.	The actively variable transmission (AVT), used along with Utah knee is redesigned to encompass stair climbing. And three level control system was developed to adapt to various ambulation modes.	Weight, peak knee velocity in swing, knee angle, torque, and power trajectories during stair climbing.	It was the lightest powered knee prosthesis that offered required torque and power for stairs climbing and level ground walk as well.
Murabayashi et al., 2022 [32]	1	To design a transfemoral prosthetic knee which can restrict undesired knee flexion during running stance phase.	Kinematic data was acquired through a three-dimensional motion system (MAC3D), and Ground reaction Forces (GRFs) were traced with three force plates (AMTI).	Prosthetic knee flexion	The designed mechanism prevented undesired flexion during stance phase; hence it could work well under normal running conditions.
Bartlett et al., 2022 [33]	1	To develop a powered knee prosthesis which provides passive torque for stance phase and support for passive swing phase with small active torques.	A novel design linear actuator was coupled with slider crank mechanism and controlled through two processors and two controllers.	Range of motion, continuous active torque	This swing-assist prosthesis increased maximum swing phase knee flexion angle; as per the varying speed; and provided accordingly supports as well.
Guercini et al., 2022 [34]	1	To develop a unique over-actuated knee prosthesis which can tackle speed and torque variation requirements for the various kinds and phases of locomotion.	This design used a dual motor approach; one was high speed/low torque motor for swing phase and other one was low dynamics high torque motor to help carrying tasks which need active torque.	Knee angle, power, torque and speed	Experimentation showed that this dual motor prosthesis resembled natural gait kinematics during level walking and produced required torque during sit-to-stand activity.
Sturk et al., 2019 [35]	20	To analyze how transfemoral amputees maintain gait symmetry over different surfaces including level, slope and uneven surfaces.	Participants walked in a virtual environment with level, inclined and uneven terrains and their various parameters were recorded for gait adaptation analysis.	Medial-lateral margin-of-stability (ML-MoS), step strategies, variability	This research provided a comprehensive evaluation and comparison of the different adaptations developed by both transfemoral amputees and healthy people over different types of surfaces.
Cao et al., 2018 [36]	12	To compare a novel microprocessor-controlled prosthetic knee (MPK) I-knee with non-microprocessor-controlled prosthetic knees (NMPKs) under various walking speeds.	The maximum swing flexion knee flexion and gait symmetry had been evaluated in I-Knee and NMPK case.	Peak knee flexion angle	The I-KNEE was better robust to speed variations, which supported the usage of I-KNEE as compared to NMPKs.
Lee et al., 2020 [37]	1	To design a stance-control swing-assist (SCSA) knee prosthesis, which can manage low output impedance of swing state for a passive stance-controlled microprocessor-controlled knees (SCMPK) swing state.	A feedback control system was developed for swing-phase motion, which resolved variations from the natural swing phase.	Mean knee angle, hip torque, and hip power	SCSA offered merits to SCMPK, like a peaceful operation and swing phase driven by inertia, hence enhanced swing-phase characteristics.
Murabayashi et al., 2022 [38]	1	To propose a new prosthetic knee mechanism for running.	The prosthetic knee mechanism would restrict flexion after a particular time period from the instant that prosthesis was off the ground.	Gait speed, swing time, knee angle and moment	The gait experiment results depicted the efficiency of the suggested mechanism for reliable running.
Lenzi et al., 2018 [39]	2	To develop a lighter in weight prosthetic knee unit with an exclusive hybrid actuation system that permits passive and powered functional modes.	A feedback controller was designed to control knee joint torque and position. The torque control system frequency response was analyzed in MATLAB.	Peak active torque and positive power at knee	This hybrid knee was the lightest prosthesis that could offer physiological torque and power during active stair climbing and passive walking on ground level.
Geng et al., 2021 [40]	1	To develop mechanical Knee-Ankle-Toe Active Transfemoral Prosthesis (KATATP) to evaluate the kinematics and dynamics features of the joints.	Mathematical modeling was done for kinematics analysis and for different gait phases analysis. Motor simulation program was developed to generate required torque.	Hip, knee, ankle and toe angles, drive torque	This model could assist amputee acquire more symmetrical walking patterns. Additionally, the plantar pressure data of the prosthesis side mimicked healthy side.
Wang et al., 2022 [41]	5	To investigate biomechanical traits of human knee joint.	Motion acquisition and evaluation system along with three 3D force measuring plates were deployed to acquire camera position by gathering the marker movement data.	Knee angle, moment, foot pressure and ground reaction force	First maximum peak value of torque was during first 25% of the gait cycle and second peak value reached in next 65%of the gait cycle. When the ankle joint moved in plantar flexion, the ground reaction force increased and finally quickly dropped to zero when the toe was off the ground,
Rahmi et al., 2022 [42]	4	To compare two kinds of prosthetic knee joints regarding their efficiency in minimizing the energy cost for walking.	A comparative quantitative research method was used by computing the average Physiological Cost Index (PCI) on each of the prosthetic knee joints.	Energy and walking speed	The outcomes proved that prosthetic knee joint four bar linkage pneumatic system showed reduced energy cost and increased walking speed, in comparison to mechanical one.
Hood et al., 2022 [43]	1	To develop adaptive control knee prosthesis for stair climbing with different stairs heights, cadences and gait patterns.	For swing phase a position controller was designed to provide required knee and ankle joint angles based on subject’s thigh movement.	Thigh orientation, knee angle, and ankle angle	This swing controller allowed stairs ascent with various heights, cadence and gait patterns by intrinsically harmonizing with the user’s thigh movements.
Cortino et al., 2022 [44]	1	To design a stair climbing controller driven by amputee’s remnant thigh movement.	A novel phase variable, merged with virtual constraints derived from healthy subject’s stair kinematics, facilitated the subject to climb stairs in a normative, step-over gait.	Phase variable and knee position	This controller facilitated active knee-ankle prostheses to execute net positive mechanical work to support stair climbing.
Hood et al, 2022 [45]	1	To present a case study with bilateral transfemoral amputations offering a pair of lightweight active knee and ankle prostheses for ground level and stair ascent.	Kinematic and kinetic evaluation quantified dissimilarities between active and passive prostheses during walking regarding three features: controlled weight acceptance, forward propulsion, and swing clearance.	Hip, knee and ankle position, knee and ankle torque and power.	This research ensured ameliorated movement and standard of life for bilateral transfemoral amputees, through active knee and ankle prostheses.
Azimi et al., 2021 [46]	3	To implement three different controllers on transfemoral prosthesis walking.	The stability of all three controllers was verified using the Lyapunov stability theorem, validating convergence to the desired gait in walking.	Knee position, velocity and torque	All three designed controllers ensured prosthetic knee tracking performance and humanlike walking for uneven surfaces.
Cheng et al., 2022 [47]	10	To develop active prosthesis control by modeling lower-limb joint kinematics for ramp walking and stair climbing, including steady-state and transitional gaits.	Both the steady-state models featured human ambulation as a function of gait phase, forward speed, and slopes, while both the transition models served to fuse those two steady state models with a conditional offset.	Hip, knee and ankle joint angles	Simulation outcomes depicted the model adaptive capability to slope prediction and mode classification errors.
Hong et al., 2019 [48]	1	To design an active transfemoral prosthesis to execute natural walking on inclined surfaces devoid of any estimation of the incline ahead.	The control scheme was based on stance phase impedance control and swing phase trajectory tracking. In the impedance control scheme, properly. During the swing phase, a Proportional-Derivative (PD) controller was deployed to track the required trajectories.	Knee joint angle, knee trajectory	This control framework facilitated transfemoral prosthesis to tackle ramp walk complications in real-time.
Andrysek et al., 2020 [49]	10	To appraise the gait patterns linked with two kinds of mechanical stance control prosthetic knee units: weight-activated braking knee and automatic stance-phase lock knee.	Spatiotemporal, kinematic, and kinetic features had been acquired through instrumented gait evaluation with a unilateral transfemoral amputation.	Swing-phase duration, range of motion and anterior pelvic tilt	The longer swing-phase duration for the weight-activated braking knee might be linked with the requirement for knee unloading to commence knee flexion during gait.
Mazumder et al., 2022 [50]	1	To introduce a novel hybrid design for above knee prosthesis control.	For intermittent and continuous walking algorithms are developed to generate command signals for the ankle and knee joints.	Knee accelerations, number of steps taken, gait phase and detected mode of the prosthesis.	To follow angular velocities is feasible by relying on the gait phase data acquired and it could assist user to align one’s reaction to the reaction of the prosthesis.
Andrysek et al., 2022 [51]	17	To compare gait features for two types of friction-based swing-phase controlled prosthetic knee units, first was a constant-friction (CF) and the second one a variable cadence controller (VCC).	A 2D motion analysis set up was deployed to calculate gait parameters.	Walking velocity, swing-phase time, cadence, stride length, step length and knee flexion	VCC ameliorated various gait patterns linked with prosthetic swing-phase control including swing-phase timing and peak knee flexion angles.
Warner et al., 2022 [52]	1	To develop a powered prosthesis practicing new impedance controller model with energy regeneration.	The prosthetic knee unit was made semi-active by storing energy in and releasing from the ultracapacitors; while interacting with the human.	Knee angle, moment and power	A first ever prosthesis which could regenerate electrical energy in a powered prosthetic knee that showed self-powered functioning in a human trial.
Best et al., 2022 [53]	2	To develop a novel phase-based task adaptive walking controller that offers continuously-variable impedance control in stance and kinematic control in swing phase.	During stance, a variable impedance controller computed joint torques and during swing a proportional derivative (PD) controller tracked intended joint angle trajectories.	Knee angle and torque	The continuous adaptive nature of this prosthesis made it preferable, as it did not represent distinct variation in behavior with minor changes in task inputs.
Gupta et al., 2019 [54]	15	To develop continuous terrain identification method for lower limb based on single channel Electromyogram deploying a simple classifier.	Support Vector Machine (SVM), Linear Discriminant Analysis (LDA) and Neural Network (NN) classifiers were used to improve average identification accuracies.	Identification accuracy for terrain, precision and sensitivity	The proposed terrain identification approach enhanced the control system efficiency, which in turn ameliorated mobility and amputees’ quality of life.
Schulte et al., 2022 [55]	10	To inspect three different model types to predict knee torque in non-weight-bearing position.	The first model comprised a convolutional neural network (CNN), second utilized a neuro-musculoskeletal model (NMS) and third model (hybrid) deployed CNN along with NMS components; all mapped EMG to knee torque; directly or indirectly.	Knee torque	Regarding error rate, CNNs efficiency was best for multi-day torque prediction.
Bittibssi et al., 2022 [56]	1	To design a learned neural network algorithm relying on recurrent neural network (RNN) for surface electromyography (sEMG) powered prosthesis actuation (PPA) system.	Three benchmark datasets were used to describe different subjects’ performance s gait patterns to construct neural network to decrease model errors in a real-time set up.	Knee joint angle	The proposed neural proved to be anticipative model for a broad variety of transfemoral prostheses control systems, and acquired excellent outcomes through hyper-parameter optimization.
Zhang et al., 2019 [57]	1	To develop an optimal design of six-bar mechanism knee joint deploying genetic algorithm.	Dynamic inverse calculation of the optimized six-bar knee prosthesis was performed through the gait data of normal people.	Knee flexion angle, knee torque	The simulation results validated good gait through this six-bar prosthetic knee.
Yang et al., 2019 [58]	12	To develop a novels EMG-based multi-feature extraction and anticipative framework to estimate knee joint angle.	The root–mean–square (RMS), wavelet coefficients (WC), and permutation entropy (PE) as characteristics of sEMG were acquired. The back propagation neural network, generalized regression neural network, and least-square support vector regression machine (LS-SVR) were utilized as anticipative framework.	Knee joint angle	The grouping of the three parameters (RMS, WC, and PE) and LS-SVR proved efficient for the knee joint angle of all types of leg movements.
Chen et al., 2022 [59]	5	To design a robust gait phase prediction method utilizing a cohesive version of piecewise monotonic gait phase thigh angle models for different ambulation modes.	A Kalman filter-based smoother was developed to fix the alteration of predicted gait phase. Relying on the suggested gait phase anticipation method, a gait phase-based joint angle following controller was developed for above knee prosthesis.	Knee joint angle	This method could attain high gait phase prediction accuracy in different ambulation modes, comprising switching modes, which had never been evaluated in other anticipation models.
Anil et al., 2022 [60]	2	To develop a control model comprising impedance check and trajectory tracking, with the changeover between the two strategies.	A PD controller was designed to develop impedance check in stance phase and trajectory tracking in swing phase.	Knee joint angle, stiffness, damping and torque	The observed kinematic and kinetic patterns with the ramp inclination were likely to ones observed in natural walking.

## Data Availability

Not applicable.
